# Cytoplasmic Synthesis in Nerve Cells

**DOI:** 10.1038/bjc.1955.72

**Published:** 1955-12

**Authors:** G. Causey, H. Hoffman

## Abstract

**Images:**


### CYTOPLASMIC SYNTHESIS IN NERVE CELLS.

G. CAUSEY AND H. HOFFMAN.

*From the Department of Anatomy, Royal College of Surgeons of England, London, W.C.2.*

Received for publication August 12, 1955.

THE chromatolytic cycle occurring in the nerve cell body (perikaryon) following removal of most of the cytoplasm by section of the nerve processes has been studied by a host of workers during the past century. The use of chromatolysis to map out the topographical distribution of nuclei and their connnections does not directly concern us here ; rather are we concerned with the parallel study of the detail of the cytological changes during the chromatolytic cycle. This concerns us firstly as an approach to the general problem of cytoplasmic synthesis and restitution, since here we have an unrivalled opportunity to study a cell from which the bulk of its cytoplasm has been removed ; secondly because the nerve cell body presents a specialised problem in protoplasmic synthesis ; its axoplasm is so extended, and so large in bulk, that the portion of the cell concerned with maintenance of cytoplasmic integrity is presented with a greater load than any other cell. The maintenance of the axon is of particular interest in view of the rather specialised reaction of nerve cells to infective and neoplastic processes. The effect of axon section on the cytoplasm of the neurones was described by Nissl (1892) and since that time the process of disintegration of the chromophil material of the nerve cell has been described as chromatolysis and its reconstitution as chromosynthesis.

The time of onset of chromatolysis and of recovery therefrom varies according to the type of injury, the site of injury and the type of cell involved, but the general order of these events can be summarised : latent period of 1 or 2 days before break up of the Nissl substance begins ; degradation of the Nissl substance is at a maximum between the 1st and 2nd weeks after axon interruption ; chromosynthesis begins in the 1st to 3rd week and may be completed by one month or may be delayed for several months (Bodian and Mellors, 1945).

Biochemical aspects and relationships of Nissl substance are discussed in three reviews in the Society of Experimental Biology Symposium on nucleic acids (1947) respectively by Caspersson, Hyden and Bodian. The detailed description of the structure of Nissl substance, as revealed by electron microscopy, has been given by Palade (1954). He finds that the basophilic element in cytoplasm (ergastoplasm) consists of double-walled vesicles or canals (the endoplasmic reticulum) surrounded by electron dense granules of approximately 120 A.U. diameter. Nissl substance was found to consist of vesicles with granules, together with dense aggregates of these granules in between. One clump of these constitutes a classical Nissl granule.

Mitochondria (chondriomes) have in general been considered as much more constant cellular elements, but very labile in regard to their reactions to fixatives.

The definition of a mitochondrion in classical microscopy has been based mainly on shape, size, vital staining, and reaction to fixatives. Palade's (1952*a*, 1953) electron microscopic studies, together with those of Sjöstrand and Rhodin (1953) revealed the internal structure—a double-layered limiting membrane of about 160 A.U. spacing, with internal cross membranes, usually in the plane of the short axis, also double-layered, and called by Palade the " cristae mitochondriales ". There is evidence to support the production of mitochondria from cytoplasm lacking differentiated elements, and also for their propagation by division (de Robertis, Nowinski and Saez, 1954).

The present work is concerned with the structural changes shown by electron-microscopic methods in dorsal root ganglion cells of the rat during the period of activity occurring in the weeks following section of the nerves distal to the ganglion.

### MATERIAL.

The 4th and 5th lumbar nerves were sectioned on one side of the rat, in the sciatic plexus, by a lateral dorso-lumbar approach. After periods of 4, 7, 10, 14, 21 and 32 days the animals were sacrificed and the 4th and 5th dorsal root ganglia of both operated and unoperated sides were removed for examination. Ganglia from unoperated animals were used as controls. All animals were anaesthetised with nembutal, 35 mg./kg.

The ganglia were fixed in 1 per cent osmium tetroxide, buffered to approximately pH 7·4 (Palade, 1952*b*), for 4 hours at + 4° C., embedded in butyl methacrylate (polymerised at room temperature by U.V. light) and cut at approximately 2–300 A.U. on a Cook and Perkins thermal expansion microtome. The sections were examined in a Metropolitan-Vickers E.M.4. microscope working at 50 k.V., using a 50 $\mu$ objective aperture and photographed at about 5000 × primary magnification.

### NORMAL MATERIAL.

As pointed out by Nageotte (1932) the cells in normal ganglia show considerable variation in the appearance of their cytoplasm ; this is illustrated in Fig. 1, a light-microscopic picture of a group of dorsal root ganglion cells. The ganglion was fixed in exactly the same manner as for electron-microscopy, sectioned at 5 $\mu$, the methacrylate dissolved out with carbon tetrachloride, and the section stained with cresyl violet. As may be seen, the size and density of the Nissl granules vary considerably from cell to cell. Apart from large, discrete granules, the density of background staining of cytoplasm varies considerably. A few myelinated fibres may be seen between the cells, and the closely adhering satellite cell capsule is shown.

In Fig. 2 an electron micrograph of the junctional region between four normal nerve cells is shown (magnification approximately 30 times that of Fig. 1). The shrinkage space between the cells is seen to be extremely small, and in some of the intercellular spaces (ranging from 0·25–0·05 $\mu$) collagen bundles are prominent. The relationship between the satellite cells and the variable layers of cytoplasm with which they encapsulate the nerve cells are clearly seen : the satellite cells are bounded by double-layered membranes (of somewhat more than 150 A.U. spacing) so that wherever two satellite cells meet a quadruple layering results, but it seems probable that this membrane is reduced between satellite cell and neurone ; sets of the membranes at higher magnification are shown in Fig. 3.

It will be observed that all four cells in Fig. 2 are rather similar in cytoplasmic composition ; all contain large and small aggregates of Nissl substance, while round and oval mitochondria are scattered throughout each cell. In the cell on the lower right a portion of the nucleus may be seen, with its dense nucleolus.

The fine structure of the cytoplasm in a densely granular cell is more clearly illustrated in Fig. 4. Scattered about the field are numerous formed mitochondria, showing cristae, whilst the Nissl substance varies from large, dense clumps of granules to relatively small aggregates, arranged around their central vesicles. As may be seen, granules commonly aggregate in rosettes and circlets of 4–6 in number. Vesicles are less common in the denser clumps of granules.

Typical mitochondria, of lengths from 0·5–1 $\mu$, are shown in Fig. 5, they are surrounded by a double-layered limiting membrane. Parallel double-lined cristae cross them almost perpendicular to the long axis. The spacing of laminae in limiting membrane and cristae is similar to that of the cell membrane.

The character of the cytoplasm of cells in the ganglia from the control side of operated animals was more variable than that of ganglion cells from normal animals. Such findings are not unexpected in view of the vascular changes following operation and alterations in postural activity.

### CELLS OF OPERATED GANGLIA.

In considering the changes that occur in cytoplasmic and nuclear structures after cutting the axons, a strictly chronological order will not be attempted because, as might be expected, all the cells in any one ganglion do not follow the identical cycle, nor the same time-course ; in fact some of the cells probably never recover. One such cell is shown in Fig. 6, where it will be seen that cytoplasmic elements are reduced to a minimum, and there is no evidence of that restorative activity which will later be shown to be evident in most of the cells. Such cells, presumably dying, have been rare in the material examined.

After allowing an interval of 21 days between section and sacrifice the picture seen in Fig. 7 was common, with eccentric nucleus and a nuclear membrane manifesting activity. No mitochondria or liposomes are seen, the cytoplasm being packed with large vesicles—some of which are beginning to show invagination of the limiting membranes—and Nissl substance is reappearing, mainly in the form of groups of granules around small vesicles.

The folding of the nuclear membrane in chromatolytic cells is a regular observation, and the most active phase is illustrated in Fig. 8, 9, 10, and 11 ; it is most striking after the 7th day. Fig. 8 and 9 are photographs of two sections, fairly close together, from the same cell ; they and Fig. 10 are from cells 14 days after axon section. The impression obtained from these corrugated nuclei is one of the throwing out of processes and the formation of large numbers of vesicles in relation to these processes. In Fig. 10 we see a dense nucleolus, double-layered nuclear membrane, and on two of the prominences are vesicles which seem still attached to the nuclear membrane. One of these seems to consist of a chain of vesicles, closely attached to each other and the nuclear prominence. This portion of the nuclear membrane is shown at larger magnification in Fig. 11, where another feature of such nuclear membranes becomes clear ; the aggregation of dense material on the inner surface of the nuclear membranes, close to prominences.

In Fig. 8 and 9 there are rows of vesicles radiating out from the nuclear membrane, some of these vesicles may be seen to have a double limiting membrane, and most have a clear central part with electron dense aggregations near the surface. Many of the vesicles are apposed or attached to promontories on the nucleus, but others are associated with hollows in the nuclear membrane. Several of the vesicles may be identified in both sections (Fig. 8 and 9,), but the cytoplasmic pattern is noticeably different. Fig. 12 represents an enlarged region from Fig. 8 and shows one of the string-like attachments extending from the nuclear promontory towards a vesicle complex. This " string " is discontinuous in Fig. 9.

From 32-day material we obtained pictures suggestive of the completion of the cycle : the nuclear membrane is becoming smooth in outline, dense mitochondria are now spreading out into the cytoplasm and in between them, masses of Nissl substance are appearing (Fig. 13–16).

### MITOCHONDRIA.

In the examination of the material described above the formation of large vesicles in relation to an activated nuclear membrane has been indicated, and the occurrence of a ring of mitochondria around the nucleus has been demonstrated and interpreted as representing a stage in the recovery of the nerve cell from trauma. It would seem that there are many intermediate forms between these two stages.

Fig. 13, 14 and 15 show part of the cytoplasm of a cell 32 days after axon section, and illlustrate the many stages that occur between the large open vesicle that is seen in parts of Fig. 13 and the formation of cristae which are shown in greater detail in the enlargements of the areas A and B illustrated in Fig. 14 and 15.

The further increase in density of the newly-formed mitochondria is illustrated in Fig. 16. There are in the cytoplasm of the cell many dense bodies, still transected by numerous cristae, but with much dense material between cristae. There are a few typical mitochondria, one such being indicated in the inset, practically the whole of it is transected by cristae. Most of the mitochondria, however, have increased their overall density by the elaboration of material between the cristae. An example of this is shown in the second inset, where cristae can be seen only faintly outlined against a dense background.

The series of transitions shown could illustrate the process whereby a typical mitochondrion, with conspicuous parallel cristae and little granular substance between them, proceeds to elaborate osmiophilic material between the cristae, until the total density of the body is such as to obliterate the cristae. It would appear that the final stage in the evolution of these cytoplasmic organelles may be the formation of osmiophilic liposomes.

### DISCUSSION.

In assessing the significance of the observations that have been summarised in this paper the first point that presents itself is the problem of artefact. Sjöstrand and Hanzon (1954) in a study of the cytoplasmic inclusions in the mouse pancreas were not able to show any definite change in the ultrastructure following the injec-

### DESCRIPTION OF PLATES.

Fig. 1.—An optical photograph of a group of normal ganglion cells, stained with cresyl violet. Note the variation both in background staining and Nissl granules in the different cells. In the capsule of the large cell on the upper right a myelinated fibre can be seen.

Fig. 2.—Electron micrograph of a junctional region between four normal ganglion cells : all four cells are rich in formed mitochondria and Nissl substance. Closely adherent to each nerve cell is the cytoplasm of the satellite cells. In the lower left centre the point of contact between several satellite cells is to be seen : this is shown in further detail in Fig. 3.

Fig. 3.—A magnified view of a group of cell membranes from Fig. 2. Note the multiple sets of double-layered membranes, with spacing between the membrane lamellae of about 160–180 A.U.

Fig. 4.—A field of typical cytoplasm in a normal ganglion cell : on the right is dense Nissl substance, composed of small groups of granules, the individual members of which are around the 120 A.U. diameter range. In the lower part of the field, and on the left, there are smaller aggregations of granules, sometimes arranged around vesicles and typical mitochondria.

Fig. 5.—Typical mitochondria from a ganglion cell. These range from 0·25 to 1 $\mu$ in their long axis, and are transected approximately perpendicular to it by double-layered cristae. The spacing in the layers of these cristae is about 150 A.U.

Fig. 6.—A very abnormal cell from a ganglion removed 10 days after operation. The nucleus can be seen surrounded by cytoplasm almost completely emptied of its organelles.

Fig. 7.—A cell from a ganglion 21 days after operation. The nuclear membrane is still somewhat folded and the cytoplasm is packed with simple, hollow vesicles. Some of these are still in close association with the nuclear membrane, but the larger vesicles are becoming separated by small vesicles and their associated rings of tiny granules. A little material may be seen within the vesicles and some of them are commencing invagination.

Fig. 8 and 9.—These two figures are photographs at the same magnification of similar regions in two serial sections of the same cell. The nuclear membrane is folded. In Fig. 8 a strand of material extends from a triangular projection on the left towards a complex group of small vesicles ; this is shown enlarged in Fig. 12. In Fig. 9 this strand is absent. Some of the hollow vesicles in Fig. 8 may also be identified in Fig. 9. A small vesicle may be seen closely adherent to a hollow in the nuclear membrane in Fig. 9 : ( ↑ ) this vesicle may also be identified in Fig. 8. The mass of the cytoplasm of this cell is packed with hollow vesicles ; no Nissl substance can be seen. From a ganglion 14 days after section of the nerve roots.

Fig. 10.—Portion of a cell from 14-day operated material. The nuclear membrane is seen to be markedly folded and from two of the projections, vesicles appear to be separating off. In these two regions the vesicles are still attached to the nuclear membrane. Throughout the cytoplasm chains of vesicles of various sizes may be seen ; in some of them the two layers of membrane are visible. The spacing of the layers is about 150 A.U.

Fig. 11.—An enlarged view of portion of the nuclear membrane, and associated vesicles, from Fig. 10. The double layering of the nuclear membrane and the attachment of the vesicles is shown. Dense aggregation can be seen on the nuclear membrane.

Fig. 12.—An enlarged portion of Fig. 8. The string-like prolongation of the nuclear membrane ( ↑ ) is seen to extend to a tightly associated group of vesicles.

Fig. 13.—Portion of a cell from 32-day material. All stages between hollow double-layered vesicles and fully formed mitochrondia may be observed. The small vesicles and associated granules and dense granular aggregations pack the spaces between the large vesicles and mitochondria.

Fig. 14.—A higher magnification of portion of Fig. 13. Here an early stage in invagination in a vesicle may be seen in the upper centre ; two cristae extend into the lumen. In the upper right is a simple vesicle, while in the lower left is an almost typical mitochondrion with short cristae.

Fig. 15.—A second magnified portion from Fig. 13. Approximately in the centre is a half-formed mitochondrion, with three regions remaining electron-transparent. Numerous cristae transect it. To the left is another body undergoing invagination, with several short invaginating buds invading a hollow centre.

Fig. 16.—Portion of a cell from 32-day material. A mitrochondrial ring in the perinuclear position is seen, but the component organelles are spreading out into the peripheral cytoplasm and some Nissl substance is appearing between them. Although the cristae are still clearly visible in many of the organelles, their overall density is increasing from laying down of material between the cristae. This is shown by comparing the body seen at *a* (enlarged in inset *a′*) where the cristae are clearly defined, with that seen at *b* (enlarged in inset *b′*) in which the cristae may be faintly detected against a dense background.

1

3

2

4

5

6

Causey and Hoffman.

7

Causey and Hoffman.

8

9

10

11

12

Causey and Hoffman.

13

14

15

Causey and Hoffman.

16

Causey and Hoffman.

tion of pilocarpine or after starvation, and draw attention to the drastic structural changes following improper fixation. We have found reproducible changes in ganglion cells after section of the nerve roots. Whilst we agree that very careful fixation is an essential it does seem legitimate to consider that with such fixation, making due allowance for variation between different specimens and checking these against the appearances in different parts of the same specimen, it is justifiable to draw conclusions from the changes in what must at worst be the same artefact, i.e. the artefact produced by buffered osmium tetroxide, or at best to consider this fixative as giving a fair reproduction of the structure in the living cell.

Of the three main structures discussed the nucleus and mitochondria are in accord with the phase contrast appearances in living cells and the Nissl substance fits well with the optical picture. The more general features of the chromatolytic cycle, such as eccentricity of the nucleus and gross depletion of a few cells, is again shown in this investigation. As the section of the nerve roots was close to the ganglia it would be expected that the changes are of a marked type (Heidenhain, 1911). The actual changes in cell size have not been studied.

The Nissl substance can be seen to go through a cycle of changes that is apparently similar to the changes recorded optically, but the individual Nissl granule of the optical picture is the aggregated clump of granules of macromolecular size. The loss of these aggregates is first seen in relation to the areas around the nuclear membrane and the dispersion of the larger aggregates such as are seen in Fig. 2, 4 and 6. It seems probable that this loss of aggregation is comparable to the stage of " dust like " particles in the classical optical picture. In material 32 days after section there is evidence of the reformation of the aggregates, but it is difficult to assess their origins. Many of the large vesicles that are found in association with the activated nuclear membrane show areas of granular and small vesicle clumps around their surfaces which might be the precursors of the Nissl material. In the description of the material two major classes of vesicle have been distinguished. The small vesicle, associated with the Nissl substance, having a diameter ranging around 500 A.U. to 1000 A.U., and the large vesicle with a diameter between 0·25 and 1·0 $\mu$ which has been associated with the mitochondrion. The relationship between the two groups is uncertain.

The mitochondria can be traced through a fairly continuous series of characteristic forms. The mitochondria of mammalian nerve cells after injury are said to be relatively stable in structure (Clarke, 1914 ; Strongman, 1917 ; McCann, 1918), but Luna (1913) described mitochondrial changes during the chromatolytic cycle in the toad. In our examination of the mitochondria they show marked changes in structure during the activity induced by section of the cell processes. It is not possible in electron microscopic studies to classify these inclusions according to their affinity for vital dyes, but the ability to define the internal structure makes it reasonable to accept the description and definition of " normal " mitochondria given by Palade (1952*a*)—the elongated body with transverse cristae. The evidence is that there are all sorts of intermediate forms between the " normal " mitochondrion defined above and the large double walled vesicles.

The internal structure of the mitochondrion was described in detail by Palade (1952*a*), who regarded the cristae mitochondriales as an infolding of the inner of the two dense laminae of the mitochondrial membrane. Sjöstrand and Hanzon (1954) do not accept the interpretation offered by Palade, they conclude that the membranes inside the mitochondrion are " individual structures with only topographic relations to the outer membrane ". Our material supports the interpretation of Palade ; the first evidence of cristae in a vesicle appears as infoldings around the periphery of the large vesicles ; the cristae seem to be associated with the inner dense layer of the surface, but our evidence on this point is not critical.

Considerable interest attaches to the relationship between the group of large vesicles and the nuclear membrane. Those cells that show the most cytoplasmic change after section of the nerve processes also show folding of the nuclear membrane. The appearances of the nuclear membrane are those of activity. There is a bulging-out of the membrane into hillocks with intervening valleys that is particularly well shown in the serial pictures. In relation to these evaginations there are in many cases rows of large vesicles. It would seem that the large vesicles are either pseudopodial processes that have been constricted off or are at least structures formed in close association with an activated nuclear membrane. Many of these vesicles show connections with the promontories on the nuclear membrane. That these vesicles then form condensations within their walls and invaginations of their wall to form a typical mitochondrion seems indicated. It is possible that further deposition of electron dense material lead to the formation of a typical liposome.

The absence of Nissl substance and mitochondria from the cytoplasm of nerve cells may be noted in a small proportion of the cells of normal ganglia, as pointed out earlier. This loss of Nissl substance and mitochondria is, however, so common in the middle period of chromatolysis as to affect the majority of the cells and gives an electron micrographic picture that may be considered equivalent to the classical chromatolytic picture of optical microscopy. There is evidence that practically all the enzymes of the cytochrome-cytochrome oxidase system are concentrated in mitochondria (Hogeboom, Claude, and Hotchkiss, 1946) and the work of Howe and Mellors (1945) established a fall in cytochrome oxidase in spinal cord during the chromatolytic period. Therefore the fate of the mitochondria of nerve cells during early stages of chromatolysis is of especial interest : if such bodies are formed from (or at) the nuclear membrane, it follows that they constitute one of the elements of the axon or dendrite which must be supplied from the perikaryon. It is possible that in the period immediately following section the mitochondria, and other formed elements of the cell body, are poured out into the cell process. The few depleted cells presumably represent individuals which have lost their organelles and are incapable of restitution.

The increasing density of certain of the mitochondria appears to be due to the laying down of electron-dense granular material between the cristae. That this material owes its density to osmiophilia is corroberated by the fact that thicker sections, cut from the same ganglion; are seen in the light microscope to possess typical osmiophil spheres in the same justanuclear position as these typical liposomes seen in the electron microscope. Practically every such body still betrays its origin in electron micrographs by the presence of dense cristae, faintly outlined against the surrounding material.

Most of the stages in the formation of vesicles and mitochondria, so evident in the operated ganglia, may be detected to a lesser degree in normal ganglia. Many simple vesicles, and various stages in the invagination to form mitochondria, have been observed. This would suggest that the processes of cytoplasmic synthesis and restitution are proceeding at various rates in the cells of normal ganglia, and that the response to axon section is merely an acceleration of this process. Our observations on Nissl substance, mitochondria and the nuclear membrane strongly support Bodian's (1947) statement: "Regeneration after interruption of the adult axon can be viewed not only as a reversion to embryonic activity but also as an artificially accelerated normal function."

### SUMMARY.

1. The electron microscopic appearances of the dorsal root ganglion cells of the rat are described and illustrated.

2. The cytoplasmic changes during the cycle of chromatolysis and chromosynthesis are described.

3. During the cycle activation of the nuclear membrane is seen and the formation of vesicular structures that can be traced in different cells through stages of incomplete crista formation to formed mitochondria and probably from formed mitochondria to liposomes.

4. The disintegration of Nissl substance has been followed and its reappearance observed.

5. The relation of these observations to the possible course of cytoplasmic synthesis in nerve cells is discussed.

This work was carried out with the financial support of the British Empire Cancer Campaign.

### REFERENCES.

Bodian, D.—(1947) *Symp. Soc. exp. Biol.*, No. 1, p. 163.
*Idem* and Mellors, R. C.—(1945) *J. exp. Med.*, **81**, 469.
Caspersson, T.—(1947) *Symp. Soc. exp. Biol.*, No. 1, p. 127.
Clarke, E.—(1914) *J. comp. Neurol.*, **24**, 61.
De Robertis, E., Nowinski, W. W. and Saez, F. A.—(1954) 'General Cytology.' Philadelphia and London (W. B. Saunders & Co.). 2nd ed., pp. 123–4.
Heidenhain, M.—(1911) 'Plasma und Zelle.' Jena (G. Fischer).
Hogeboom, G. H., Claude, A. and Hotchkiss, R. D.—(1946) *J. biol. Chem.*, **165**, 615.
Howe, H. A. and Mellors, R. C.—(1945) *J. exp. Med.*, **81**, 489.
Hyden, H.—(1947) *Symp. Soc. exp. Biol.*, No. 1, p. 152.
Luna, E.—(1913) *Anat. Anz.*, **44**, 413.
McCann, G. F.—(1918) *J. exp. Med.*, **27**, 31.
Nissl, F.—(1892) *Allg. Z. Psychiat.*, **48**, 197.
Nageotte, J.—(1932) 'Cytology and Cellular Pathology of the Nervous System.' New York (Hoeber). Vol. 1.
Palade, G. E.—(1952*a*) *Anat. Rec.*, **114**, 427.—(1952*b*) *J. exp. Med.*, **95**, 285.—(1953) *J. Histochem. Cytochem.*, 1, 188.—(1954) *Congr. int. Microscop. électron.* (in press).
Sjöstrand, F. S. and Hanzon, V.—(1954) *Exp. Cell Res.*, **7**, 393.
*Idem* and Rhodin, J.—(1953) *Ibid.*, **4**, 426.
Strongman, B. T.—(1917) *Anat. Rec.*, **12**, 167.


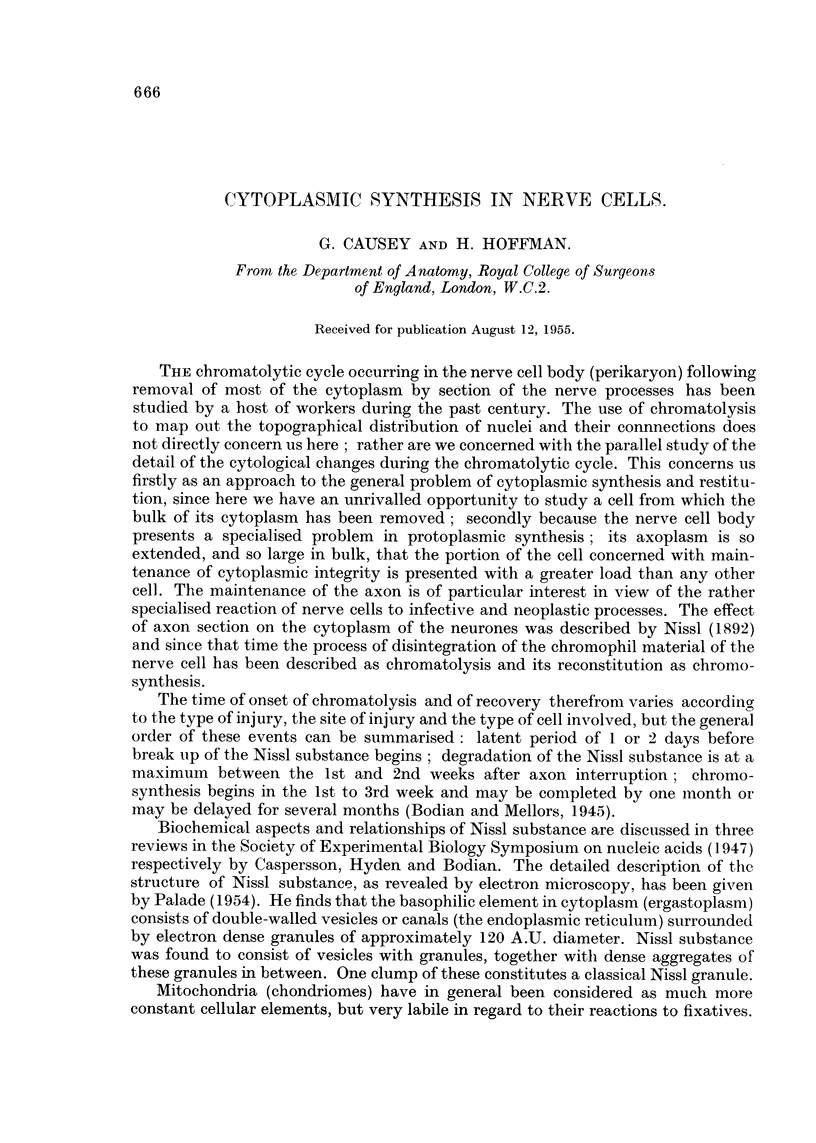

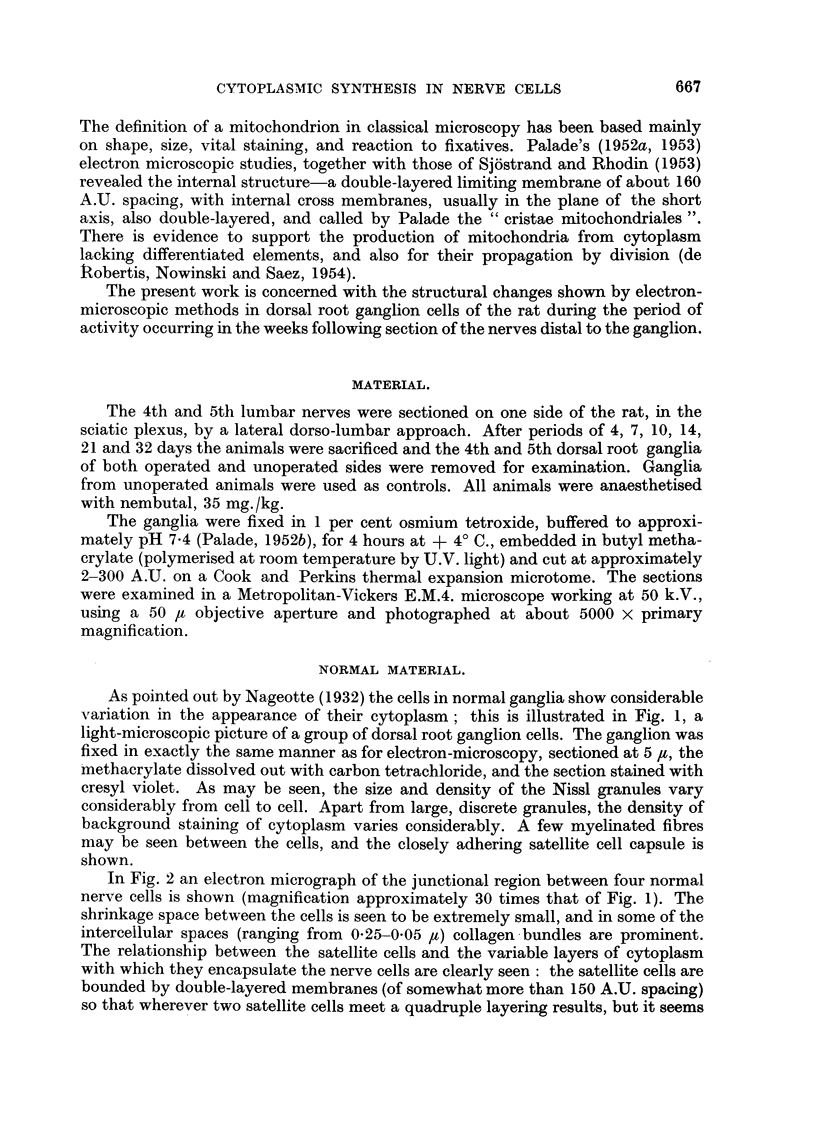

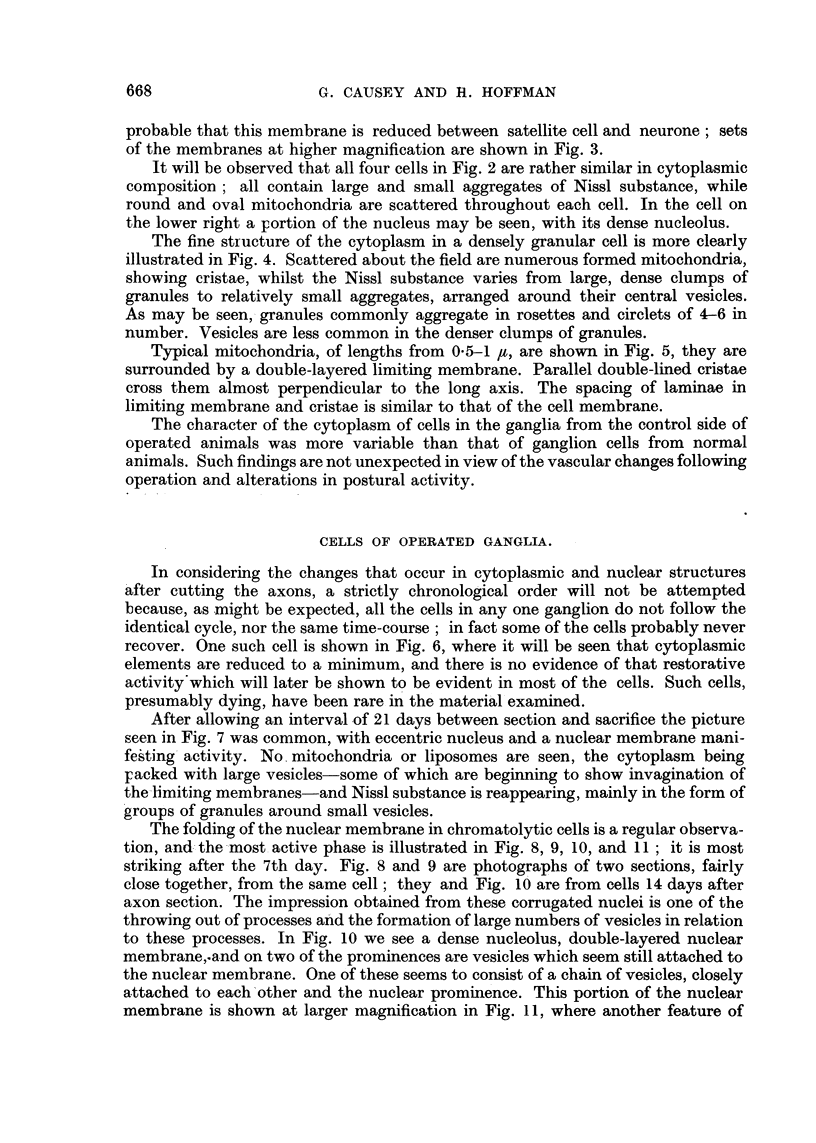

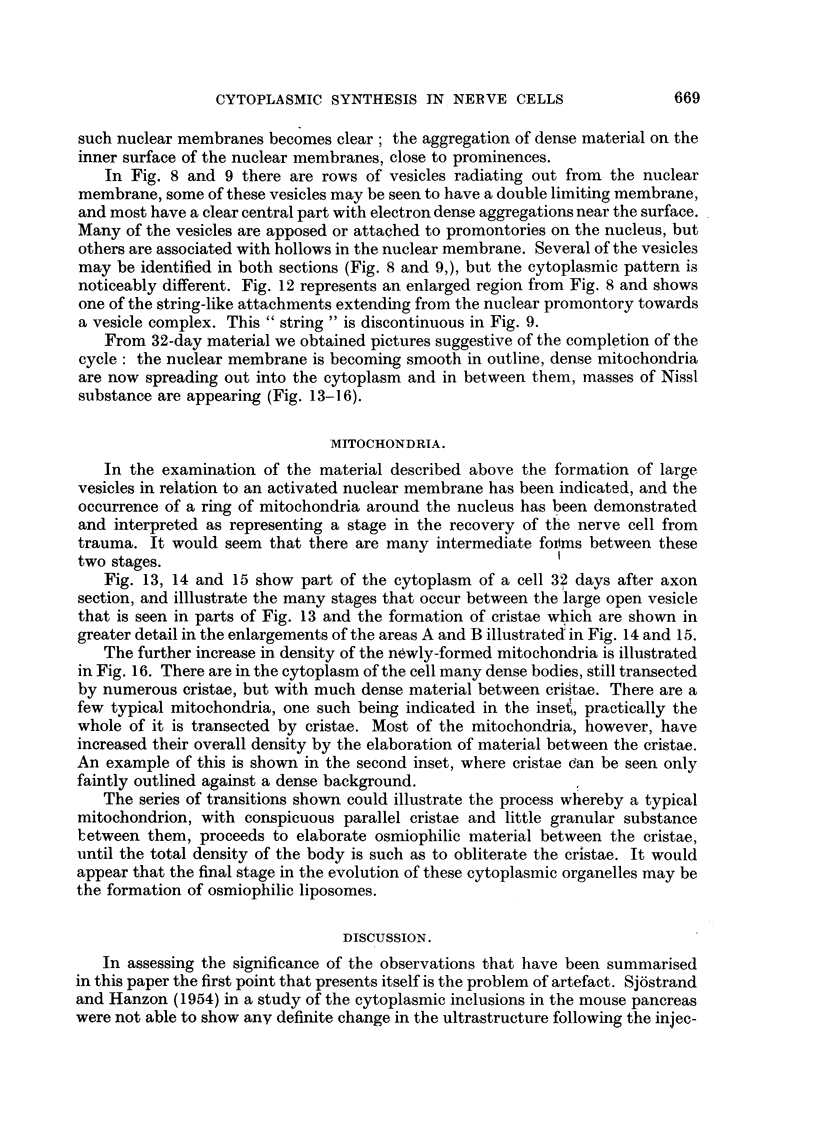

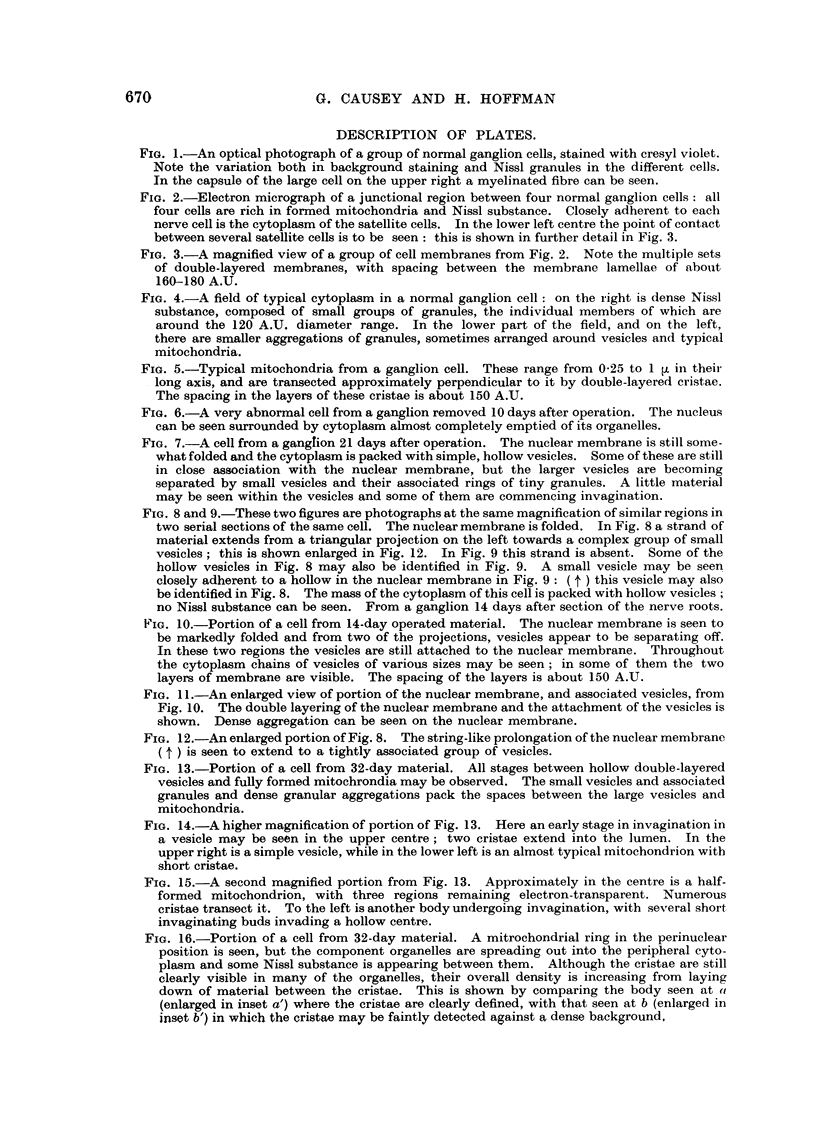

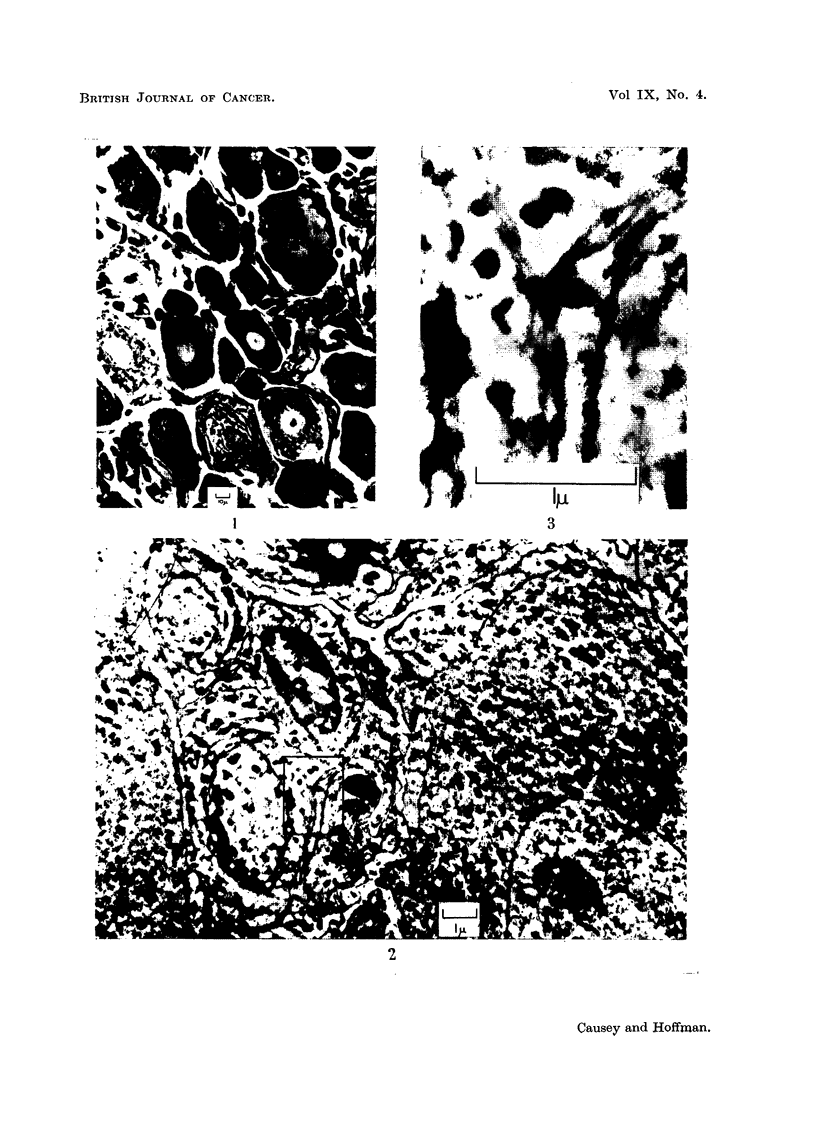

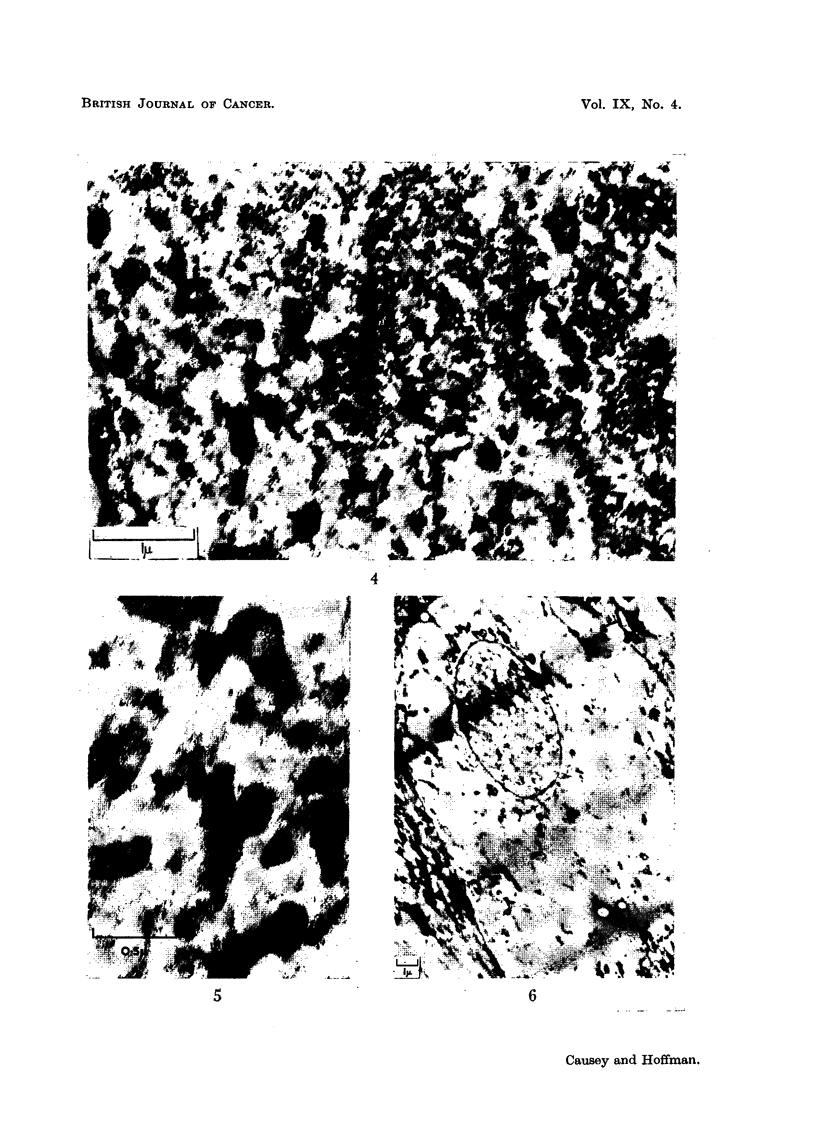

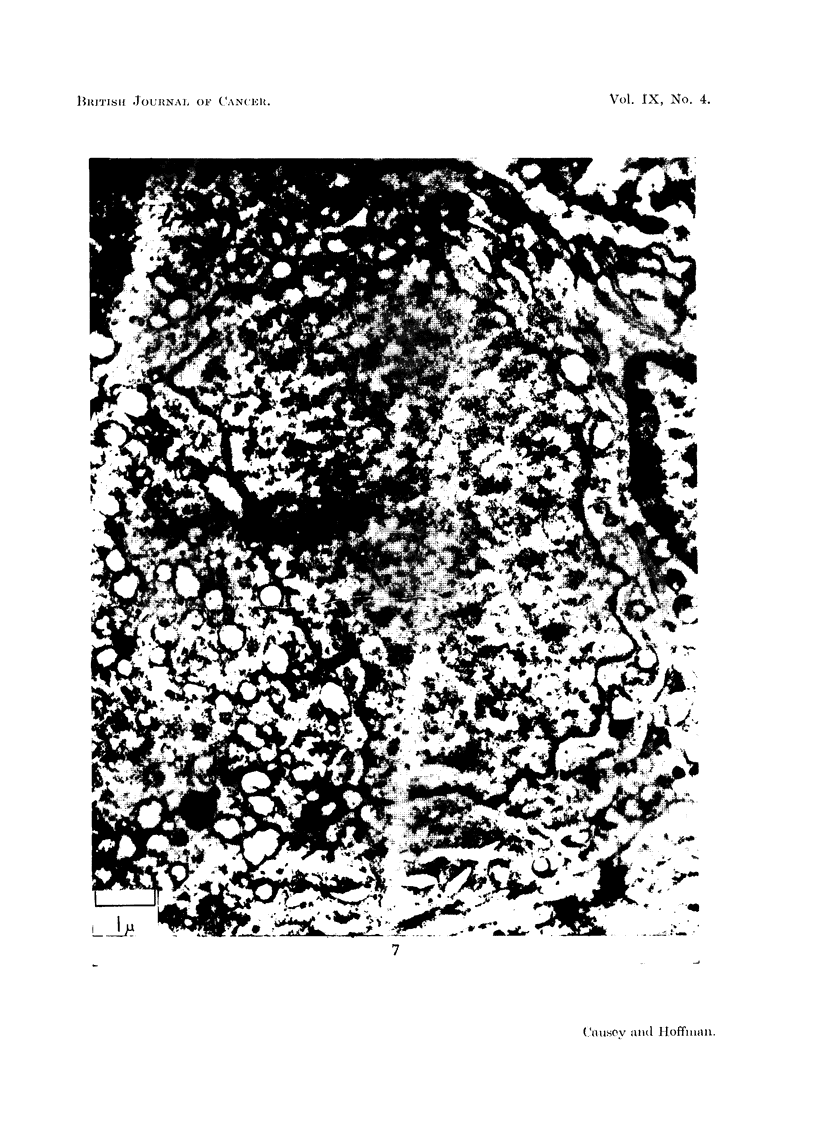

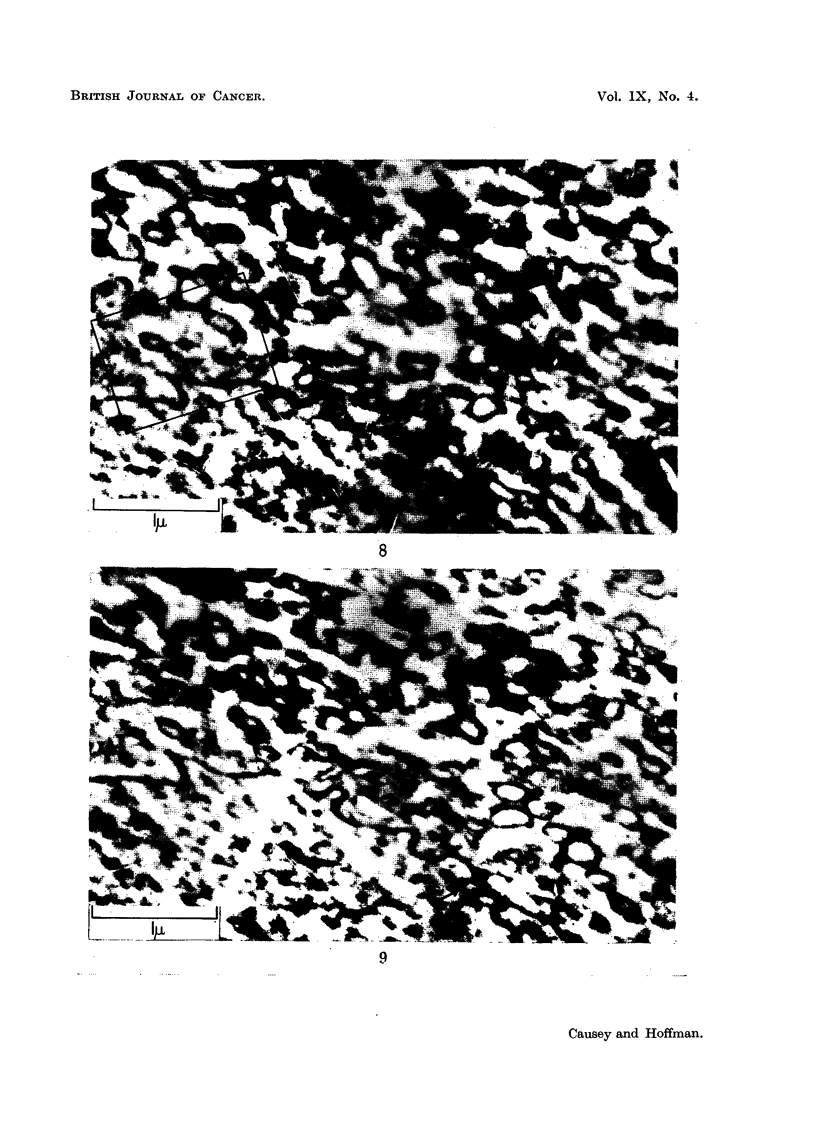

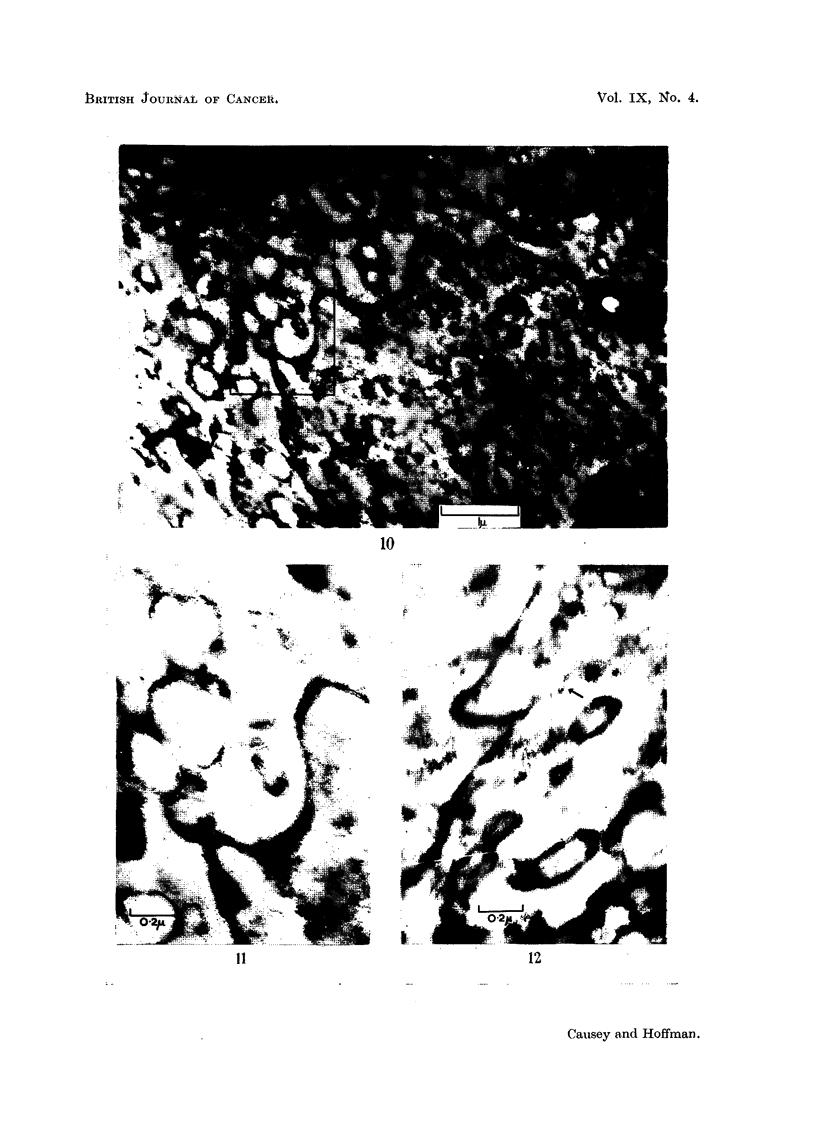

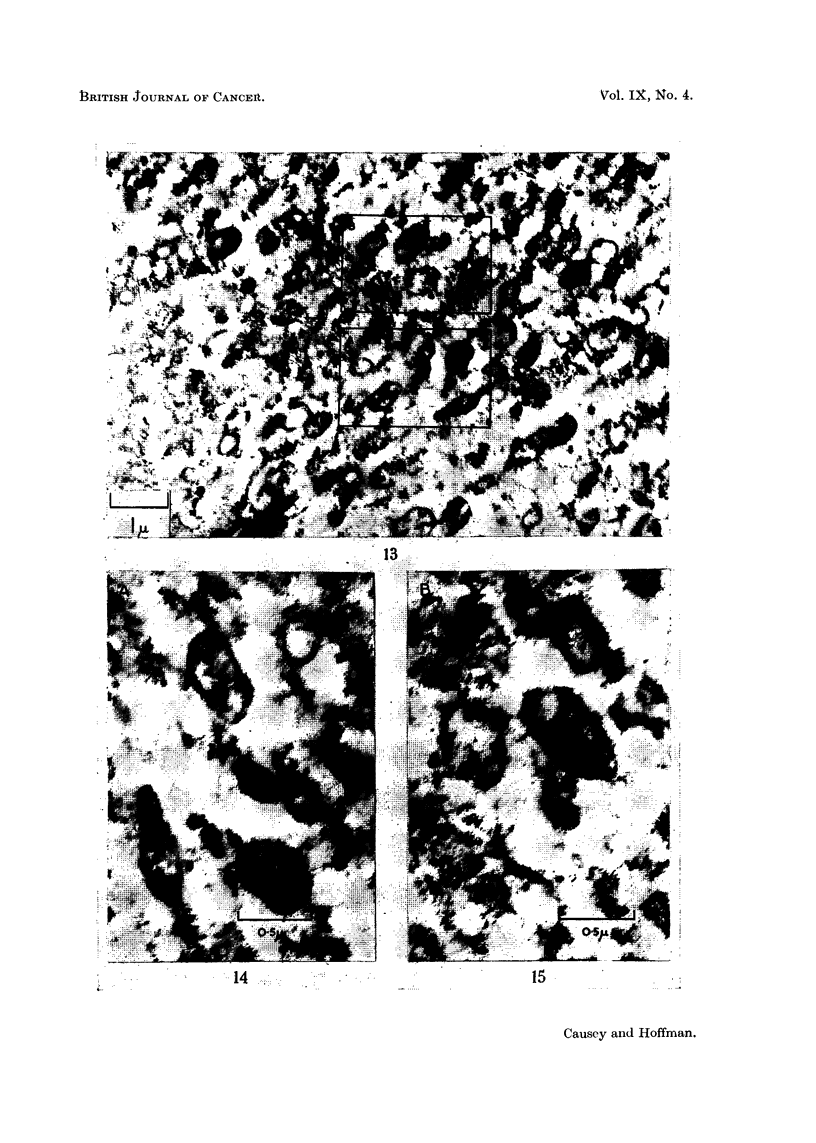

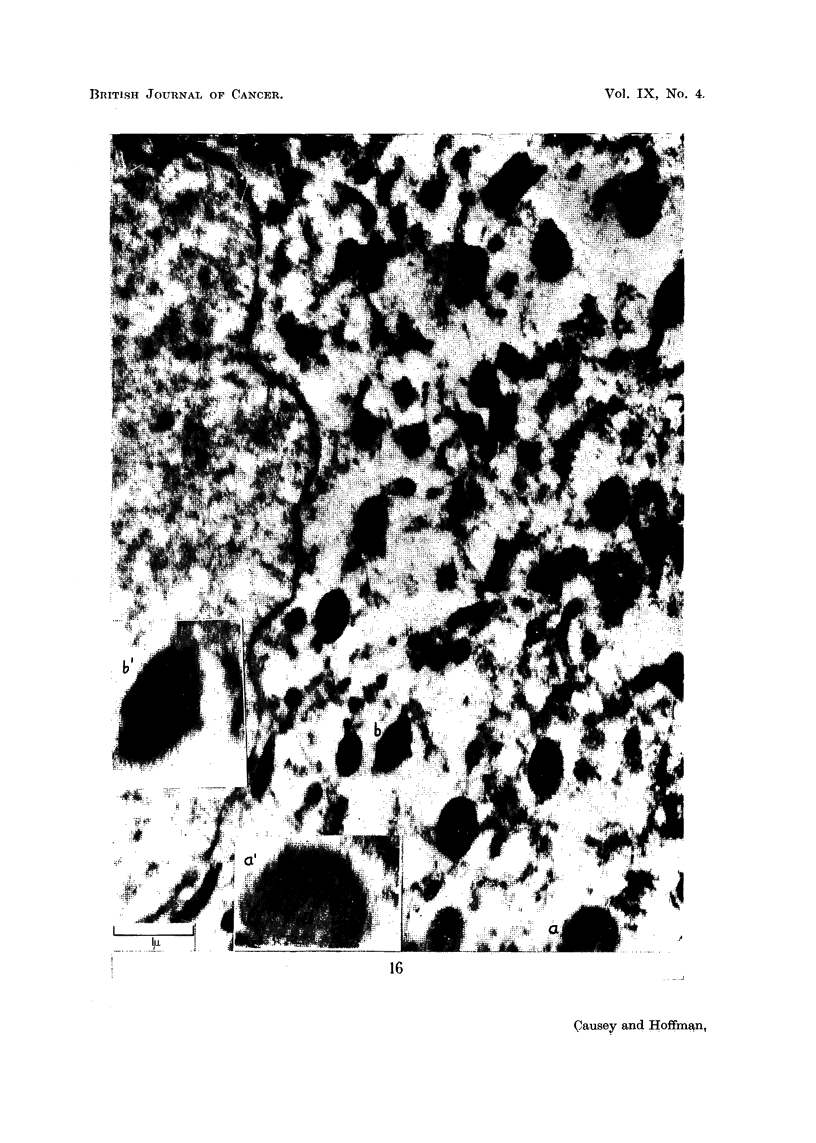

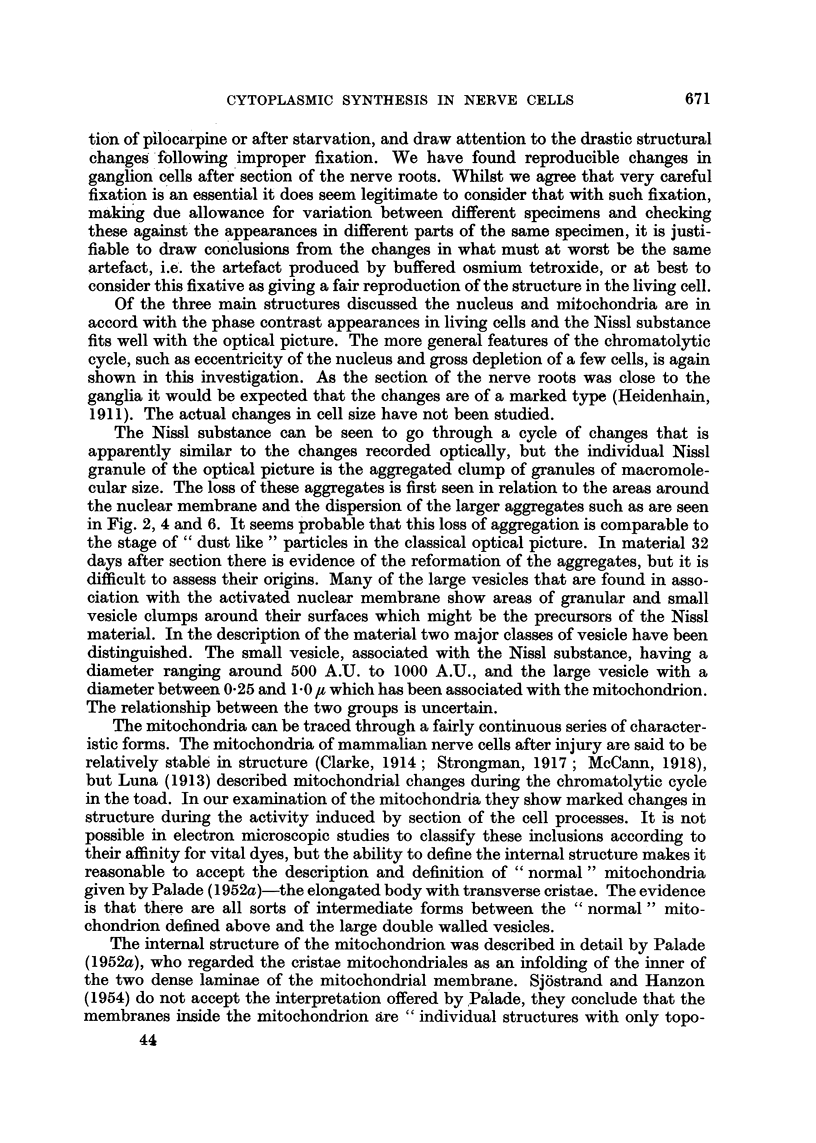

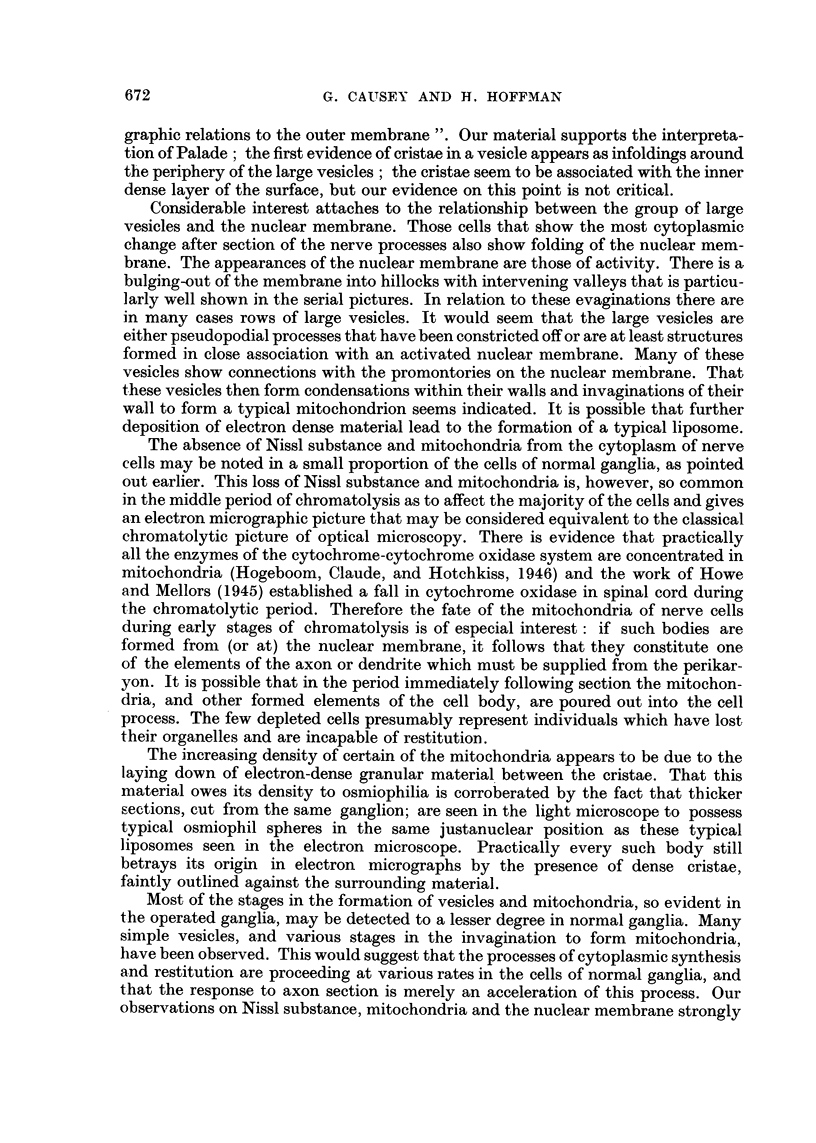

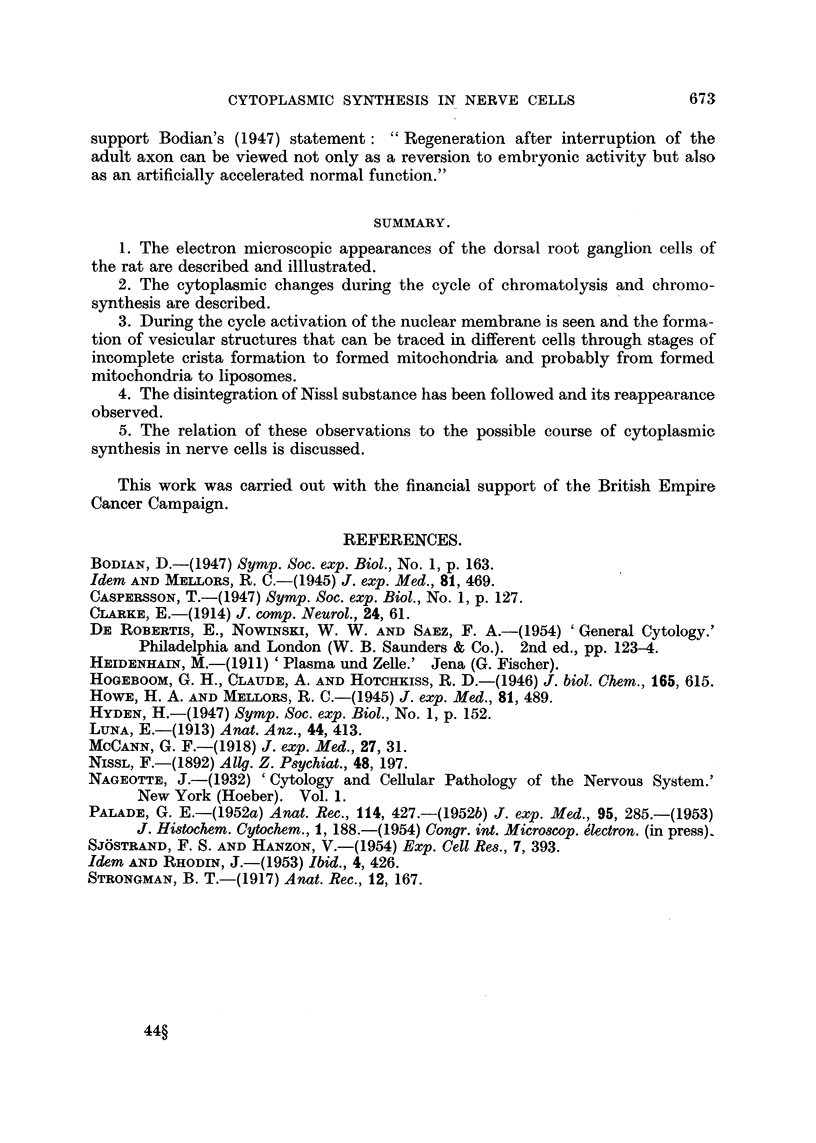


## References

[OCR_00661] PALADE G. E. (1953). An electron microscope study of the mitochondrial structure.. J Histochem Cytochem.

[OCR_00664] SJOSTRAND F. S., HANZON V. (1954). Membrane structures of cytoplasm and mitochondria in exocrine cells of mouse pancreas as revealed by high resolution electron microscopy.. Exp Cell Res.

